# Plastome phylogenomics of *Saussurea* (Asteraceae: Cardueae)

**DOI:** 10.1186/s12870-019-1896-6

**Published:** 2019-07-02

**Authors:** Xu Zhang, Tao Deng, Michael J. Moore, Yunheng Ji, Nan Lin, Huajie Zhang, Aiping Meng, Hengchang Wang, Yanxia Sun, Hang Sun

**Affiliations:** 1 CAS Key Laboratory of Plant Germplasm Enhancement and Specialty Agriculture, Wuhan Botanical Garden, Chinese Academy of Sciences, Wuhan, 430074 Hubei China; 2 University of Chinese Academy of Sciences, Beijing, 100049 China; 3 Key Laboratory for Plant Diversity and Biogeography of East Asia, Kunming Institute of Botany, Chinese Academy of Sciences, Kunming, 650201 Yunnan China; 4 Department of Biology, Oberlin College, 119 Woodland St, Oberlin, OH USA

**Keywords:** *Saussurea*, Rapid radiations, Plastome, Genome, Phylogenomic analysis, Purifying selection

## Abstract

**Background:**

*Saussurea* DC. is one of the largest and most morphologically heterogeneous genera in Asteraceae. The relationships within *Saussurea* have been poorly resolved, probably due an early, rapid radiation. To examine plastome evolution and resolve backbone relationships within *Saussurea*, we sequenced the complete plastomes of 17 species representing all four subgenera.

**Results:**

All *Saussurea* plastomes shared the gene content and structure of most Asteraceae plastomes. Molecular evolutionary analysis showed most of the plastid protein-coding genes have been under purifying selection. Phylogenomic analyses of 20 *Saussurea* plastomes that alternatively included nucleotide or amino acid sequences of all protein-coding genes, vs. the nucleotide sequence of the entire plastome, supported the monophyly of *Saussurea* and identified three clades within it. Three of the four traditional subgenera were recovered as paraphyletic. Seven plastome regions were identified as containing the highest nucleotide variability.

**Conclusions:**

Our analyses reveal both the structural conservatism and power of the plastome for resolving relationships in congeneric taxa. It is very likely that differences in topology among data sets is due primarily to differences in numbers of parsimony-informative characters. Our study demonstrates that the current taxonomy of *Saussurea* is likely based at least partly on convergent morphological character states. Greater taxon sampling will be necessary to explore character evolution and biogeography in the genus. Our results here provide helpful insight into which loci will provide the most phylogenetic signal in *Saussurea* and Cardueae.

**Electronic supplementary material:**

The online version of this article (10.1186/s12870-019-1896-6) contains supplementary material, which is available to authorized users.

## Background

*Saussurea* DC. is one of the largest genera in the family Asteraceae [1, 2]. It comprises approximately 300 species that are distributed in Asia, Europe and North America, with the highest diversity in the Himalayas and central Asia [3, 4]. *Saussurea* exhibits extreme morphological diversity and exists in habitats ranging from steppes to moist forests to cold and dry alpine meadows above 5000 m [3, 5].

Several phylogenetic studies have been conducted on *Saussurea* but the circumscription and infrageneric relationships of the genus remain controversial [5–12]. Lipschitz [13] recognized a total of 390 species belonging to six subgenera, namely subg. *Saussurea* DC., *Jurinocera* (Baill.) Lipsch., *Eriocoryne* (DC.) Hook. f., *Amphilaena* (Stschegl.) Lipsch., *Theodorea* (Cass.) Lipsch. and *Frolovia* (DC.) Lipsch. However, molecular evidence [7, 10, 14] has indicated that subg. *Jurinocera*, subg. *Frolovia* and sect. *Elatae* of subg. *Saussurea* should be excluded from *Saussurea* and treated as independent genera: *Lipschitziella* R.V. Kamelin, *Frolovia* (DC.) Lipsch. and *Himalaiella* Raab-Straube, respectively. Using molecular and morphological evidence, Shi and Raab-Straube [3] suggested *Saussurea* sect. *Aucklandia* should be treated as a new genus, *Aucklandia* Falc. Based on sequences of five loci (*rbcL, ndhF, matK, trnL-F* and ITS) and morphology, Wang et al. [11] established the genus *Shangwua* Y. J Wang, Raab-Straube, Susanna & J. Q. Liu from sect. *Jacea*, leaving four subgenera (*Saussurea, Eriocoryne, Amphilaena* and *Theodorea*) as constituting *Saussurea* s.s. Despite this progress, the relationships among and within these four subgenera have been poorly resolved due to a potentially rapid radiation, leaving insufficient phylogenetic signal at deeper nodes [5]. No phylogenomic studies have yet assessed these relationships, although a recent study using target enrichment of nuclear genes to resolve Cardueae relationships sampled 19 species of *Saussurea* representing two subgenera [15].

Plastomes have been proven to be powerful tools for exploring deep relationships in the plant Tree of Life [16–19]. They have helped resolve ambiguous relationships of particularly recalcitrant lineages, such as those that have undergone rapid evolutionary radiations (e.g. [20–23]). Complete plastome sequences also provide insight into the molecular evolutionary patterns associated with gene rearrangements, duplication and loss (e.g. [24–26]), and in some cases these structural changes are phylogenetically informative characters in and of themselves, as for example the two large inversions (~ 20 kb and ~ 3 kb inversions) that characterize the Large Single-Copy (LSC) region of most Asteraceae plastomes [27–29].

Different regions of the plastome have different selective constraints that may yield differing estimates of phylogeny, as for example noncoding versus coding regions [30, 31]. Selective forces may also play a role in driving plastome structure [32], including rearrangements [33] and gene loss [34, 35]. However, the effect of selective forces in plastome evolution within Asteraceae remains unclear.

To date, only three *Saussurea* plastomes have been reported: *S. involucrata* [36], *S. chabyoungsanica* [37] and *S. polylepis* [38]. Here, we sequenced 17 species representing all four subgenera of *Saussurea* in order to (1) elucidate plastome evolution, including structural variation and molecular signals of selection, (2) estimate the effectiveness of different plastome data sets in resolving relationships within this radiating lineage, and (3) investigate the backbone relationships within *Saussurea*.

## Results

### Characteristics of *Saussurea* plastomes

**Fig. 1:**
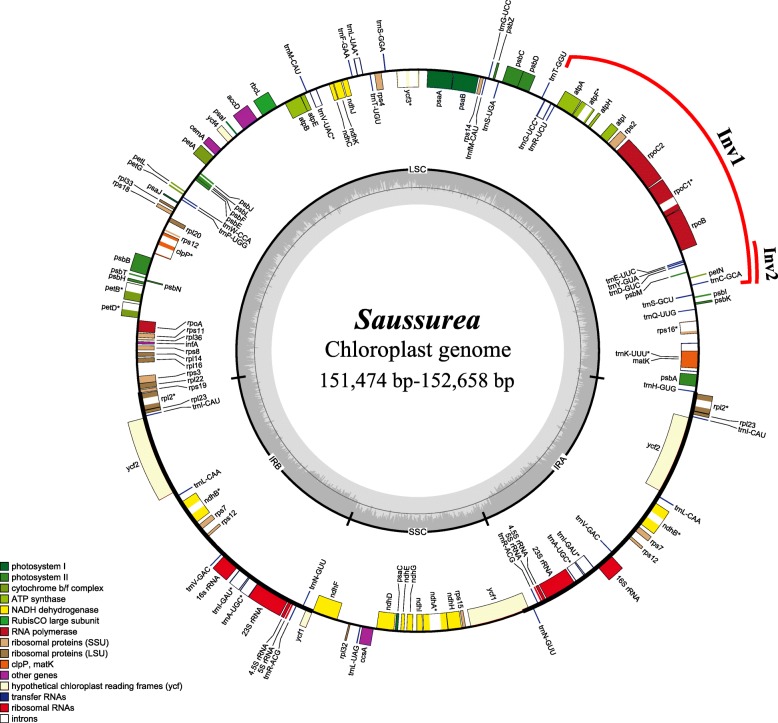
General plastome map of *Saussurea.* Specific sizes for the plastomes of each species are presented in Table 1. Inv: inversions

After de novo and reference-guided assembly, we obtained a single scaffold for each plastome. The sequencing and assembly information are provided in Tables 1 and Additional file 1: Table S2. The sizes of the 17 *Saussurea* plastomes were similar, ranging from 151,474 bp in *S. tridactyla* to 152,658 bp in *S. przewalskii*. All 17 plastomes possessed the typical angiosperm quadripartite structure and contained 113 unique genes, including 79 protein-coding genes, 30 transfer RNA (tRNA) genes and four ribosomal RNA (rRNA) gene. A total of 18 genes (including 11 protein-coding genes and 7 tRNA genes) had introns, with 15 genes having one intron and three genes having two introns. The IR regions were also highly consistent, all of which included 17 genes (six protein-coding genes, seven tRNA genes, and four rRNA genes). In all plastomes, the *rps12* gene was found to be trans-spliced, with one of its exons located in the LSC region and the other duplicated in the IR (Fig. 1).

**Table 1:** Characteristics of newly sequenced plastomes

Species	Plastome size (bp)	LSC length (bp)	IR length (bp)	SSC length (bp)	Gene Number	Protein coding genes	RNAs	GC content (%)
*Saussurea hookeri* C.B.Clarke	152,461	83,437	25,201	18,622	114	80	34	37.7
*Saussurea obvallata* (DC.) Sch.Bip.	152,544	83,460	25,196	18,692	114	80	34	37.7
*Saussurea pubifolia* S.W.Liu	152,622	83,541	25,227	18,627	114	80	34	37.7
*Saussurea* sp. nov	152,055	83,543	24,660	19,192	114	80	34	37.7
*Saussurea psudoleucoma* Y. S. Chen	152,412	83,450	25,183	18,596	114	80	34	37.7
*Saussurea lhozhagensis* Y.S.Chen	152,527	83,525	25,194	18,614	114	80	34	37.7
*Saussurea gossypiphora* D.Don	152,463	83,451	25,199	18,614	114	80	34	37.7
*Saussurea tridactyla* Sch.Bip. ex Hook.f.	151,474	82,564	25,193	18,524	114	80	34	37.7
*Saussurea gnaphalodes* (Royle) Sch. Bip.	152,475	83,379	25,201	18,694	114	80	34	37.7
*Saussurea salwinensis* J.Anthony	152,382	83,449	25,194	18,545	114	80	34	37.7
*Saussurea przewalskii* Maxim.	152,658	83,533	25,221	18,683	114	80	34	37.7
*Saussurea delavayi* Franch.	152,254	83,213	25,196	18,649	114	80	34	37.7
*Saussurea leontodontoides* (DC.) Sch.Bip.	152,387	83,433	25,201	18,552	114	80	34	37.7
*Saussurea durgae* C. Jeffrey & R.C.Srivast.	152,506	83,411	25,195	18,705	114	80	34	37.7
*Saussurea kingii* C.E.C.Fisch.	152,444	83,398	25,178	18,690	114	80	34	37.6
*Saussurea japonica* (Thunb.) DC.	152,612	83,502	25,140	18,830	114	80	34	37.7
*Saussurea tsoongii* Y.S.Chen	152,501	83,373	25,193	18,742	114	80	34	37.7

The ~ 20 kb and ~ 3 kb inversions (Inv1 and Inv2) of most Asteraceae were detected in all *Saussurea* plastomes (Fig. 1). Inv1 was located between the *trnG-UCC* and *trnS-GCU* genes; Inv2 (located between the *trnS-GCU* and *trnE-UUC* genes) was nested within the large inversion and shared one end-point with Inv1 (Fig. 1). Sliding window analysis showed much higher proportions of variable sites in single-copy regions than in the IR regions. Seven relatively highly variable regions (*rps16-trnQ*, *trnS-trnC-petN, psbE-petL, ndhF-rpl32, rpl32-trnL, rps15* and *ycf1*) were identified from the plastome sequences (Fig. 3).

### Selection analyses

Most protein coding genes showed a low dN/dS ratio (ω; Additional file 2: Figure S1), indicating that they have been under purifying selection. Only three genes (*psbL, psbZ* and *ycf2*) had ω > 1, but the branch model results revealed no significant difference between foreground and background branches (Table 2).

**Table 2:** Branch model results of three genes with ω > 1. ω = dN/dS, the ratio of nonsynonymous/synonymous substitution rates. np: number of parameters. ln L: log likelihood values. LRT: likelihood ratio test

Genes	Model	np	ln L	Estimates of parameters		LRT *P* value
*psbL*	Two ratio Model 2	67	−177.133731	ω_0_ = 2.64875	ω_1_ = 1.71333	0.9984
	Model 0	66	−177.133729	ω_0_ = 2.64876		
*ycf2*	Two ratio Model 2	67	−10,731.616197	ω_0_ = 1.39172	ω_1_ = 1.98555	0.7353
	Model 0	66	−10,731.67334	ω_0_ = 1.40184		
*psbZ*	Two ratio Model 2	67	− 1424.307323	ω_0_ = 2.73007	ω_1_ = 2.21091	0.9972
	Model 0	66	−1424.307317	ω_0_ = 2.73007		

### Phylogenetic analyses

Our phylogenomic analyses substantially increased resolution and provided robust backbone relationships of *Saussurea* (Fig. 4, Additional file 3: Figure S2). Characteristics of the three concatenated data sets are presented in Table 3. Dataset-3 had the highest number of parsimony-informative (PI) characters, followed by dataset-1 and dataset-2. Centaureinae were resolved as sister to *Saussurea* in datasets-1 and -3 with strong support, but not in dataset-2. All three datasets also strongly supported the monophyly of *Saussurea* (BS = 100), while three (*Eriocoryne, Amphilaena,* and *Saussurea*) of the four traditional subgenera were resolved as paraphyletic. Three main clades of *Saussurea* were identified. Clade 1 included three species of subg. *Amphilaena* (*S. publifolia, S.* sp. nov., *S. involucrata*), one of subg. *Eriocoryne* (*S. lhozhagensis*) and five of subg. *Saussurea* (*S. durgae, S.przewalskii, S. salwinensis, S. delavayi, S. kingii*). Clade 2 included two species of subg. *Amphilaena* (*S. hookeri, S. obvalata*) and four of subg. *Eriocoryne* (*S. gnaphalodes, S. gossypiphora, S. psedoleucoma,* and *S. tridactyla*). Clade 3 included two species of subg. *Theodorea* (*S. japonica* and *S. tsoongii*) and two Korean species (*S. chabyoungsanica, S. polylepis*). Datasets-1 and -2 resolved subg. *Theodorea* as sister to remaining *Saussurea*, whereas dataset-3 resolved clade 2 as sister to remaining *Saussurea*, albeit with low support (Fig. 4, Additional file 3: Figure S2). The coalescent-based result yielded an almost identical topology with the concatenation-based phylogeny (dataset-1), except for the position of *S. kingii*, which was resolved as sister to clade 1 + clade 2 (Additional file 4: Figure S3).

**Table 3:** Characteristics of the three different data sets

Dataset	Composition	Total number of characters (bp)	Variable sites (bp)	Parsimony-informative (PI) sites (bp)	Singleton sites (bp)	Proportion of PI sites (%)
(1)	Nucleotide sequences of all 79 protein-coding regions (CDS)	69,474	4071	2285	1782	3.29
(2)	Amino acid sequences of all 79 CDS (AA)	23,158	1835	1179	653	5.09
(3)	Complete plastome nucleotide sequences	134,955	10,622	5490	5043	4.07

## Discussion

### Plastome evolution

**Fig. 2:**
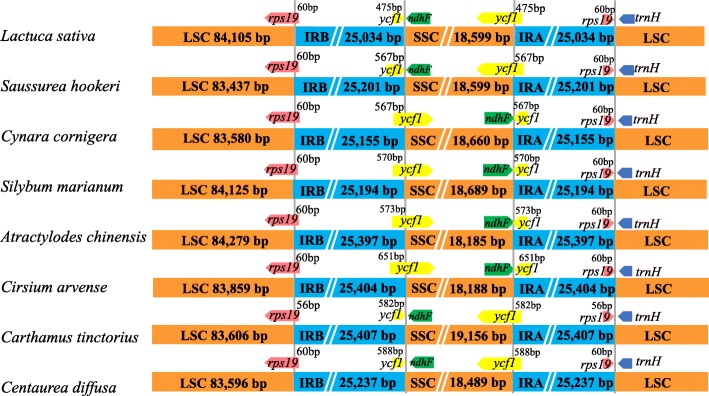
Comparison of the LSC, IR and SSC borders among seven Cardueae genera, with *Lactuca sativa* as a reference

The 20 *Saussurea* plastomes in our analyses indicated that plastome evolution has been conservative within this genus. All *Saussurea* plastomes possessed the typical plastome structure of most Asteraceae, including both LSC inversions that are present in nearly all Asteraceae, as for example in *Lactuca* [27]*, Artemisia* [29]*, Lasthenia* [28]*, Taraxacum* [39] and *Mikania* [40]. The expansion and contraction of the IR region has been demonstrated to be a significant source of length variation in some plastomes, e.g. early-diverging eudicots [41, 42] and Apiales [43]. In the present study, however, no significant IR length variation was detected among *Saussurea* plastomes (Fig. 2).

In our molecular evolutionary analysis, most protein-coding genes were found to be under purifying selection (Additional file 2: Figure S1). This pattern has also been demonstrated in other Asteraceae plastomes, such as in *Mikania cordata* [40] and *Helianthus* [44], reflecting the typically conservative evolution of plastome genes in green plants. Indeed, the best evidence for relaxation of purifying selection is in plants that have lost photosynthesis, in which genes involved directly in photosynthesis evolve much faster due to loss of function, typically resulting in pseudogenization and eventual gene loss [32, 34, 35]. Nevertheless, complete genome- and transcriptome-based analyses are necessary to fully investigate the importance of selection at protein-coding loci in plastids, given that most plastid proteins are encoded in the nucleus.

### Phylogenetically informative sites

**Fig. 3:**
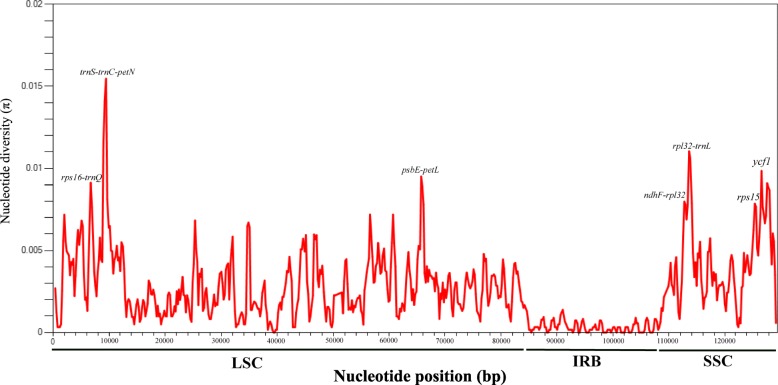
Sliding window analysis of nucleotide variability (Pi) across 31 complete plastome sequences of Cardueae, with one copy of the IR included

To resolve relationships among closely related species, it is imperative to identify rapidly evolving loci. Previous phylogenetic studies of *Saussurea* mainly favored three plastid loci (*trnL-F*, *psbA-trnH,* and *matK*) but these have failed to resolve relationships across the genus (e.g. [5–8]). Our analyses revealed relatively low nucleotide diversity in these three regions (Fig. 3), explaining the low resolution in previous analyses and highlighting the importance of exploring more of the plastome to obtain additional informative sites and regions. We found seven relatively variable regions: *rps16-trnQ*, *trnS-trnC-petN, psbE-petL, ndhF-rpl32, rpl32-trnL, rps15* and *ycf1.* Of these, *rps16-trnQ*, *trnC-petN*, *psbE-petL, rpl32- trnL*, *rps15* and *ycf1* have been previously reported as hotspots of divergence and have been broadly used for reconstructing phylogeny in plant taxa [40, 45–50]. The lineage-specific, rapidly evolving regions identified here will facilitate further phylogenetic resolution of the large and diverse *Saussurea*.

### Phylogenetic relationships within *Saussurea*

**Fig. 4:**
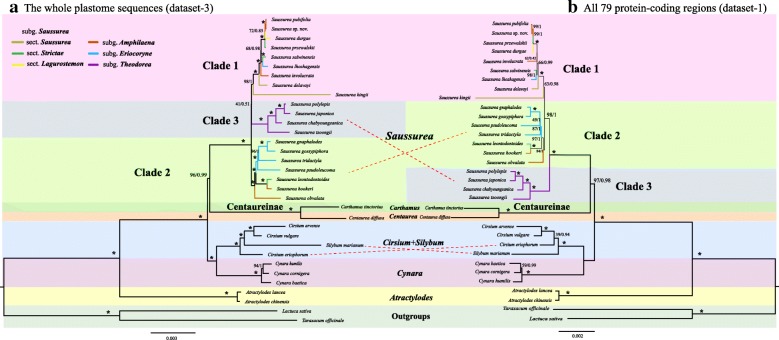
Inferred molecular phylogeny from ML (maximum likelihood) and BI (Bayesian inference) analyses using different data sets. **a** Complete plastome sequences (dataset-3); **b** All 79 CDS (dataset-1). Maximum likelihood bootstrap values (BS) and posterior probabilities (PP) are shown at nodes. Branches with * have 100% bootstrap support

The backbone relationships of *Saussurea* have been poorly resolved in previous molecular phylogenetic studies (e.g., [5–8, 10–12]). Our analyses greatly increased resolution with generally robust support (Fig. 4, Additional file 3: Figure S2). With the exception of subg. *Theodora* (the only monophyletic subgenus), there is relatively little concordance between the relationships recovered here and morphological characters used to define sections and subgenera [3, 4, 13]. In fact, these morphological characters have been shown to have adaptive value, as for example the dense woolly trichomes and colorful bracts that are used to circumscribe subg. *Eriocoryne* and subg. *Amphilaena* respectively. These two kinds of character states are prevalent among alpine species, and have been thought to protect plants from cold and UV-B radiation at high elevations [5, 51–53]. Hence, the discordance between phylogeny and morphology may reflect potential convergent evolution in *Saussurea*. It is also important to note that our estimate of phylogeny is based only on the plastome in a rapidly radiating group. Given that incomplete lineage sorting (ILS) or hybridization are most likely to obscure the species phylogeny among close relatives, it is possible that the addition of nuclear phylogenomic data may result in a different estimate of relationships in *Saussurea*. Consequently, it is essential to expand taxon and locus sampling significantly within *Saussurea* to better understand patterns of character state evolution and biogeography.

The clades formed by subg. *Theodorea* and sect. *Laguranthera* (*S. durgae*) were resolved as early-diverging groups in phylogenetic studies of *Saussurea* based on ITS and *trnL-trnF* [7]. In our concatenated datasets-1 and -2 and coalescent-based approach, the early-diverging position of subg. *Theodorea* was also supported, despite it being relatively distant phylogenetically from sect. *Laguranthera*. Across all concatenated datasets, *S. kingii* had the longest branch by far (Fig. 4, Additional file 3: Figure S2), which was also detected in the phylogenetic study of Wang and Liu [12]. As suggested there, this likely results from its biennial habit, as substitution rates are known to be higher in species with shorter generation times [54]. In addition, the systematic position of *S. kingii* was unstable between concatenated- and coalescent-based approaches, suggesting a further investigation may be required.

Incongruence at deeper levels among the trees resulting from our three concatenation-based analyses is likely related to differences in the number of parsimony-informative (PI) characters among data sets, with the highest number of PI characters in dataset-3 (Table 3). These differences likely explain the better overall support for the backbone of *Saussurea* in the tree based on dataset-3 (Fig. 4a) compared to the other trees. Given the relatively low taxonomic level (within a genus) of our study, it makes sense that including nucleotide sequence, especially for noncoding regions, would maximize the power to resolve relationships. We therefore recommend complete plastome data sets in these situations. The incongruence at a few backbone nodes is not surprising given how short these branches are; it is likely that few PI characters ever existed at these branches, and hence such nodes are sensitive to the conditions of phylogenetic analysis [23, 55].

## Conclusions

Our analyses reveal both the structural conservatism and power of the plastome for resolving relationships in congeneric taxa. By examining signals of selection at protein-coding loci, we are able to eliminate systematic error due to selective biases as a source of topological incongruence. Hence, it is very likely that differences in topology among data sets are due primarily to differences in numbers of parsimony-informative characters. Our study further demonstrates that currently accepted subgeneric groups in *Saussurea* are likely based at least partly on convergent character states, and are therefore in need of revision. Moreover, greater taxon sampling is necessary to disentangle the patterns of character evolution and biogeography that are only hinted at here. Our results here provide helpful insight into which loci will provide the most PI sites in *Saussurea* and Cardueae, but they also suggest that complete plastome sequencing will be a valuable technique for resolving the relationships in this difficult genus.

## Methods

### Taxon sampling, chloroplast DNA isolation, high-throughput sequencing

We sequenced 17 new plastomes representing 16 currently described and one undescribed species of *Saussurea*; collection and voucher information are provided in Additional file 1: Table S1. These were added to the three previously reported plastomes available in GenBank (Additional file 1: Table S1). The circumscription and infrageneric treatment of *Saussurea* followed *Flora of China* and *Flora of Pan-Himalaya* [3, 4]. For all species, total DNA was extracted from fresh or silica gel-dried leaves with a modified CTAB (Cetyl trimethylammonium bromide) method [56]. Sequencing libraries were constructed and quantified following the methods introduced by Sun et al. [41]. For all plastomes, a 500-bp DNA TruSeq Illumina (Illumina Inc., San Diego, CA, USA) sequencing library was constructed using 2.5–5.0 ng sonicated DNA as input. Libraries were quantified using an Agilent 2100 Bioanalyzer (Agilent Technologies, Santa Clara, CA, USA) and by real-time quantitative PCR. Libraries were then multiplexed and sequenced using a 2 × 125 bp run on an Illumina HiSeq 2000 platform at Novogene in Kunming, Yunnan, China.

### Plastome assembly, annotation, and comparative analyses

Raw sequence reads were subsequently filtered using Trimmomatic v.0.36 [57] with the following parameters: SLIDING WINDOW = 4:20, MINLEN = 50, LEADING = 3, TRAILING = 3, HEAD-CROP = 12, and AVGQUAL = 20. Remaining high-quality reads were assembled de novo into contigs with a minimum length of 1000 bp using CLC Genomics Workbench 11.0 (https://www.qiagenbioinformatics.com/) with default parameters. The resulting de novo contigs were then reference-assembled against the plastome of *S. chabyoungsanica*. Finished plastomes were annotated using DOGMA [58] and GeSeq [59]. Manual adjustments of start/stop codons and intron/exon boundaries were conducted in Geneious version 9.0.5 [60], using published plastomes of *Saussurea* as references. The tRNA genes were identified with tRNAscan-SE [61]. Physical maps of the circular plastomes were visualized with OGDRAW [62].

We performed plastome comparisons between *Saussurea polylepis* and six other Cardueae genera (*Cirsium arvense, Carthamus tinctorius, Cynara cornigera, Centaurea diffusa, Silybum marianum, Atractylodes chinensis*). All seven complete plastomes were aligned with ProgressiveMAUVE [63], assuming collinear genomes for the full alignment. To assess sequence divergence and determine highly phylogenetically informative sites, nucleotide variability (π) was calculated by sliding window analysis conducted in DnaSP version 6.11.01 [64] with all aligned plastome sequences of *Saussurea*. For the purposes of alignment, the SSC region was inverted manually in Geneious as necessary. The step size was set to 200 bp, with a 600 bp window length.

### Phylogenetic analyses

Thirty-one taxa (Additional file 1: Table S1) of Cardueae (20 *Saussurea* + 11 outgroup genera from Cardueae) and two outgroup taxa of Cichorieae (*Lactuca sativa, Taraxacum officinale*) were included in phylogenetic analyses. Both concatenated and coalescent-based analyses were conducted. For concatenation-based approach, three datasets were analyzed: dataset-1 included the nucleotide sequences of all 79 protein-coding sequences (CDS); dataset-2 included the amino acid sequences of these 79 CDS; and dataset-3 included the complete plastome nucleotide sequences, including only one copy of the IR regions. Dataset-1 and -2 were created by concatenating alignments using PhyloSuite version 1.1.15 [65]. Characteristics of all three data sets were calculated using MEGA X [66]. For all concatenated data sets, Modeltest version 3.7 [67] was used to estimate the optimal model under the Akaike Information Criterion (AIC). Maximum likelihood (ML) analyses were conducted using RAxML version 8.2.10 [68] under the general time reversible model of nucleotide substitution, with the gamma model of rate heterogeneity (GTRGAMMA for dataset-1 and daset-3; PROTGAMMAAUTO for dataset-2). Bootstrap (BS) support was estimated with 1000 bootstrap replicates using the “rapid bootstrap” algorithm of RAxML. Bayesian inference (BI) was performed using MrBayes version 3.2.3 [69]. Two runs were conducted in parallel with four Markov chains (one cold and three heated), with each running for 5000,000 generations from a random starting tree and sampled every 5000 generations. Convergence was assessed by examining the average standard deviation of split frequencies (ASDF). After ASDF reached < 0.01, the first 25% of the trees were discarded as burn-in, and the remaining trees were used to construct majority-rule consensus trees.

For the coalescent-based analysis, ML unrooted trees for 79 CDS alignments were estimated separately using RAxML under the GTRGAMMA model with 500 bootstrap replicates. ASTRAL III version 5.6.2 algorithm [70] was used to estimate the species tree from 79 gene trees with node supports calculated as local posterior probabilities.

### Analyses of signatures of selection

To test for evidence of selection in plastid protein coding genes, we estimated the ratio of nonsynonymous (dN) to synonymous (dS) substitutions (ω) for all 79 protein coding genes using CodeML in PAML version 4.9 [71] with the following settings: model = 0, seqtype =1, NSsites = 0. Genes showing higher ω were identified with the branch model [72, 73] to determine lineage-specific selection in plastomes of *Saussurea*. Following the recommendations in CodeML, the best ML tree determined by RAxML with dataset-1 using concatenation-based approach was used as the input topology, and the clade formed by *Saussurea* was set as a foreground branch. The likelihood ratio and *P* value were used to test if a model (“model = 2”) of positive selection on the foreground branch was a significant improvement over a null model (“model = 0”) where no positive selection occurred on the foreground branch.

## Additional files

Additional file 1: **Table S1.** Taxa included in present study. Collection locality and voucher information are provided for newly sequenced **Table S2.** The sequencing and assembly information of newly sequenced plastomes. Q30: the percentage of bases with Phred quality score greater than 30 in the total base.plastomes. (DOCX 27 kb)

Additional file 2: **Figure S1.** The ratio of nonsynonymous and synonymous substitutions (ω, dN/dS) within each protein coding gene, as calculated by CodeML in PAML. Genes with ω > 1 are colored in red. (PDF 1045 kb)

Additional file 3: **Figure S2.** Inferred molecular phylogeny from ML (maximum likelihood) and BI (Bayesian inference) analyses for the amino acid sequence of (79 CDS; data set 2). Maximum likelihood bootstrap values (BS) and posterior probabilities (PP) are shown at nodes. Branches with * have 100% bootstrap support and 1.0 posterior probability. (PDF 272 kb)

Additional file 4: **Figure S3.** Estimated species tree from 79 CDS alignment by coalescent-based approach. Local posterior probabilities are labeled at nodes. Branches with * have 1.0 posterior probability. The clade of *S. kingii* is colored in red. (PDF 548 kb)

## Data Availability

All sequences used in this study are available from the National Center for Biotechnology Information (NCBI) (see Additional file 1: Table S1).

## Abbreviations

AIC: Akaike Information Criterion
ASDF: Average standard deviation of split frequencies
BI: Bayesian Inference
CDS: Protein-coding sequences
CTAB: Cetyl trimethylammonium bromide
DnaSP: DNA Sequences Polymorphism
DOGMA: Dual Organellar Genome Annotator
GTR: General time reversible
IR: Inverted repeat
ITS: Internal transcribed spacer of ribosomal DNA
LSC: Large single copy
ML: Maximum Likelihood
NCBI: National Center for Biotechnology Information
PI: Parsimony-informative
rRNA: Ribosomal RNA
SSC: Small single copy
tRNA: Transfer RNA
