# Supplementary material for: Plastome phylogenomics of Saussurea (Asteraceae: Cardueae)
Source: BMC Plant Biol. 2019 Jul 2;19:290. doi: 10.1186/s12870-019-1896-6 (PMC6604455; doi:10.1186/s12870-019-1896-6)
Supplement: Supplementary file 1 — Table S1. Taxa included in present study. Collection locality and voucher information are provided for newly sequenced Table S2. The sequencing and assembly information of newly sequenced plastomes. Q30: the percentage of bases with Phred quality score greater than 30 in the total base.plastomes. (DOCX 27 kb) [file 12870_2019_1896_MOESM1_ESM.docx]

**Table S1** Taxa included in present study. Collection locality and voucher information are provided for newly sequenced plastomes.

| Taxa | Family | Genus | Collection locality | Voucher information | Herbarium information | Genebank accession number |
| --- | --- | --- | --- | --- | --- | --- |
| *Saussurea hookeri* C.B.Clarke | Asteraceae | *Saussurea* | China, Xizang, Lhünzê | ZJW5360 | KUN | MK952740 |
| *Saussurea pubifolia* S.W.Liu | Asteraceae | *Saussurea* | China, Xizang, Nang | ZJW5296 | KUN | MK953466 |
| *Saussurea* sp.nov | Asteraceae | *Saussurea* | China, Xizang, Qusum | ZJW6769 | KUN | MK953467 |
| *Saussurea psudoleucoma* Y. S. Chen | Asteraceae | *Saussurea* | China, Xizang, Lhünzê | ZJW5383 | KUN | MK953468 |
| *Saussurea lhozhagensis* Y.S.Chen | Asteraceae | *Saussurea* | China, Xizang, Lhünzê | ZJW5344 | KUN | MK953469 |
| *Saussurea obvallata (DC.) Sch.Bip.* | Asteraceae | Saussurea | China, Xizang, Lhünzê | ZJW5520 | KUN | MK953470 |
| *Saussurea gossypiphora* D.Don | Asteraceae | *Saussurea* | China, Xizang，Nyalam | ZJW6369 | KUN | MK953471 |
| *Saussurea tridactyla* Sch.Bip. ex Hook.f. | Asteraceae | *Saussurea* | China, Xizang，Nyalam | ZJW6372 | KUN | MK953472 |
| *Saussurea gnaphalodes* (Royle) Sch. Bip. | Asteraceae | *Saussurea* | China, Sichuan, Darzêdo | FSC-117 | KUN | MK953473 |
| *Saussurea salwinensis* J.Anthony | Asteraceae | *Saussurea* | China, Xizang, Lhünzê | ZJW5375 | KUN | MK953474 |
| *Saussurea przewalskii* Maxim. | Asteraceae | *Saussurea* | China, Xizang, Cona | ZJW5445 | KUN | MK953475 |
| *Saussurea delavayi* Franch. | Asteraceae | *Saussurea* | China, Yunnan, Dali | ZJW5112 | KUN | MK953476 |
| *Saussurea leontodontoides* (DC.) Sch.Bip. | Asteraceae | *Saussurea* | China, Xizang, Lhünzê | ZJW5538 | KUN | MK953477 |
| *Saussurea durgae* C.Jeffrey & R.C.Srivast. | Asteraceae | *Saussurea* | China, Xizang, Chomo | ZJW6084 | KUN | MK953478 |
| *Saussurea kingii* C.E.C.Fisch. | Asteraceae | *Saussurea* | China, Xizang, Quxur | ZJW6729 | KUN | MK953479 |
| *Saussurea japonica* (Thunb.) DC. | Asteraceae | *Saussurea* | China, Sichuan, Ruoergai Xian | KUN40249 | KUN | MK953481 |
| *Saussurea tsoongii* Y.S.Chen | Asteraceae | *Saussurea* | China, Yunnan, Dêqên Zong | MS17-700 | KUN | MK953480 |
| *Saussurea chabyoungsanica* Im | Asteraceae | *Saussurea* | - | - | - | KX622799 |
| *Saussurea polylepis* Nakai, 1931 | Asteraceae | *Saussurea* | - | - | - | MF695711 |
| *Saussurea involucrate* (Kar. & Kir.) Sch. Bip. | Asteraceae | *Saussurea* | - | - | - | KU041648 |
| *Atractylodes chinensis* (Bunge) DC. | Asteraceae | *Atractylodes* | - | - | - | MG874805 |
| *Atractylodes lancea* (Thunb.) DC. | Asteraceae | *Atractylodes* |  |  | - | MG874804 |
| *Carthamus tinctorius* L. | Asteraceae | *Carthamus* | - | - | - | NC_030783 |
| *Centaurea diffusa* Lam. | Asteraceae | *Centaurea* | - | - | - | KJ690264 |
| *Cirsium arvense* (L.) Scop. | Asteraceae | *Cirsium* | - | - | - | KY562583 |
| *Cirsium eriophorum* (L.) Scop. | Asteraceae | *Cirsium* | - | - | - | KY562584 |
| *Cirsium vulgare* (Savi) Ten. | Asteraceae | *Cirsium* | - | - | - | KY562585 |
| *Cynara baetica* (Spreng.) Pau | Asteraceae | *Cynara* | - | - | - | KP842706 |
| *Cynara humilis* L. | Asteraceae | *Cynara* | - | - | - | KP299292 |
| *Cynara cornigera* Lindl. | Asteraceae | *Cynara* | - | - | - | KP842707 |
| *Silybum marianum* (L.) Gaertn. | Asteraceae | *Silybum* | - | - | - | KT267161 |
| *Lactuca sativa* L. | Asteraceae | *Lactuca* | - | - | - | AP007232 |
| *Taraxacum officinale* F.H.Wigg. | Asteraceae | *Taraxacum* | - | - | - | KU361241 |

KUN, Herbarium, Kunming Institute of Botany, Chinese Academy of Sciences; -, Not applicable.

**Table S2** The sequencing and assembly information of newly sequenced plastomes. Q30: the percentage of bases with Phred quality score greater than 30 in the total base.

| Taxa | Raw Reads | Clean Reads | Map Reads | Q30(%) | Plastome size (bp) | Coverage (x) |
| --- | --- | --- | --- | --- | --- | --- |
| *Saussurea hookeri* C.B.Clarke | 8,332,122 | 8,323,926 | 247,754 | 92.65 | 152,461 | 243.755 |
| *Saussurea obvallata* (DC.) Sch.Bip. | 8,120,763 | 8,114,770 | 309,912 | 92.82 | 152,544 | 304.744 |
| *Saussurea pubifolia* S.W.Liu | 8,299,046 | 8,292,105 | 348,774 | 92.81 | 152,622 | 342.782 |
| *Saussurea* sp. nov | 7,296,032 | 7,290,596 | 500,353 | 91.71 | 152,055 | 493.591 |
| *Saussurea psudoleucoma* Y. S. Chen | 9,304,138 | 9,295,357 | 363,667 | 92.96 | 152,412 | 357.912 |
| *Saussurea lhozhagensis* Y.S.Chen | 8,391,353 | 8,385,052 | 491,415 | 89.64 | 152,527 | 483.273 |
| *Saussurea gossypiphora* D.Don | 8,222,114 | 8,215,779 | 181,283 | 93.05 | 152,463 | 178.354 |
| *Saussurea tridactyla* Sch.Bip. ex Hook.f. | 8,767,447 | 8,758,380 | 297,352 | 92.6 | 151,474 | 294.458 |
| *Saussurea gnaphalodes* (Royle) Sch. Bip. | 7,891,911 | 7,884,681 | 274,868 | 92.12 | 152,475 | 270.406 |
| *Saussurea salwinensis* J.Anthony | 8,851,189 | 8,844,388 | 360,219 | 92.19 | 152,382 | 354.588 |
| *Saussurea przewalskii* Maxim. | 8,699,590 | 8,691,483 | 253,097 | 92.7 | 152,658 | 248.690 |
| *Saussurea delavayi* Franch. | 8,007,150 | 8,000,291 | 150,584 | 92.6 | 152,254 | 148.355 |
| *Saussurea leontodontoides* (DC.) Sch.Bip. | 8,866,683 | 8,856,911 | 482,063 | 92.16 | 152,387 | 474.512 |
| *Saussurea durgae* C.Jeffrey & R.C.Srivast. | 8,949,857 | 8,942,403 | 273,928 | 92.65 | 152,506 | 269.427 |
| *Saussurea kingii* C.E.C.Fisch. | 9,656,482 | 9,643,216 | 535,960 | 92.43 | 152,444 | 527.367 |
| *Saussurea japonica* (Thunb.) DC. | 11,031,401 | 11,007,265 | 326,875 | 90.76 | 152,612 | 321.280 |
| *Saussurea tsoongii* Y.S.Chen | 9,260,104 | 9,240,353 | 322,391 | 90.48 | 152,501 | 317.104 |
