# Supplementary material for: Plastome phylogenomics of Saussurea (Asteraceae: Cardueae)
Source: BMC Plant Biol. 2019 Jul 2;19:290. doi: 10.1186/s12870-019-1896-6 (PMC6604455; doi:10.1186/s12870-019-1896-6)
Supplement: Supplementary file 2 — Figure S1. The ratio of nonsynonymous and synonymous substitutions (ω, dN/dS) within each protein coding gene, as calculated by CodeML in PAML. Genes with ω > 1 are colored in red. (PDF 1045 kb) [file 12870_2019_1896_MOESM2_ESM.pdf]

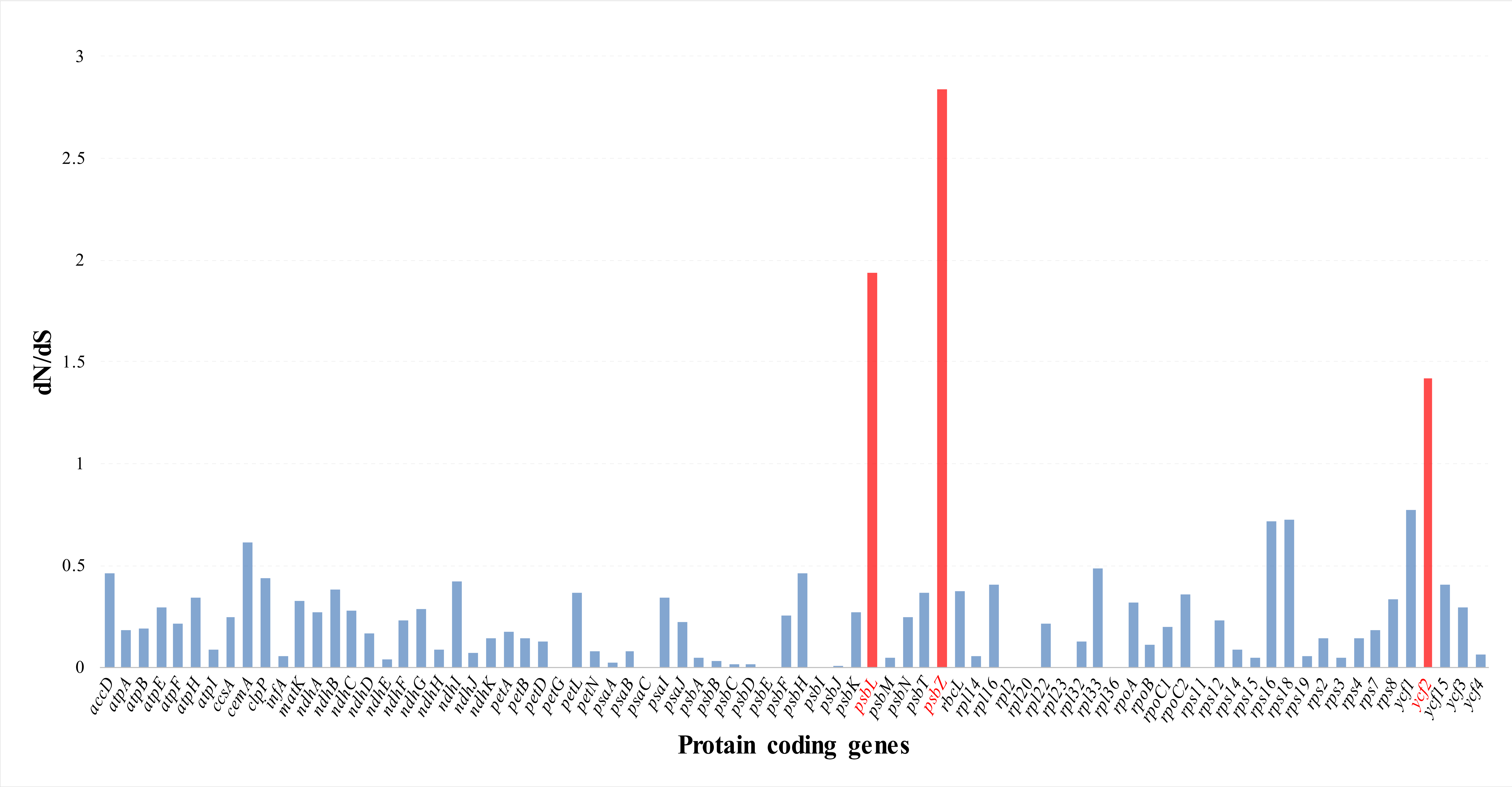
dN/dS
3
2.5
2
1.5
1
0.5
0
accD atpA atpB atpE atpF atpH atpI ccsA cemA clpP infA matK ndhA ndhB ndhC ndhD ndhE ndhF ndhG ndhH ndhI ndhJ ndhK petA petB petD petG petL petN psaA psaB psaC psaI psaJ psbA psbB psbC psbD psbE psbF psbH psbI psbJ psbK psbL psbM psbN psbT psbZ rbcL rpl14 rpl16 rpl2 rpl20 rpl22 rpl23 rpl32 rpl33 rpl36 rpoA rpoB rpoC1 rpoC2 rps11 rps12 rps14 rps15 rps16 rps18 rps19 rps2 rps3 rps4 rps7 rps8 ycf1 ycf2 ycf15 ycf3 ycf4
Protain coding genes
