# Supplementary figures and images for: Plastome phylogenomics of Saussurea (Asteraceae: Cardueae)
Source: BMC Plant Biol. 2019 Jul 2;19:290. doi: 10.1186/s12870-019-1896-6 (PMC6604455; doi:10.1186/s12870-019-1896-6)

The amino acid sequences of 79 CDS (dataset-2)

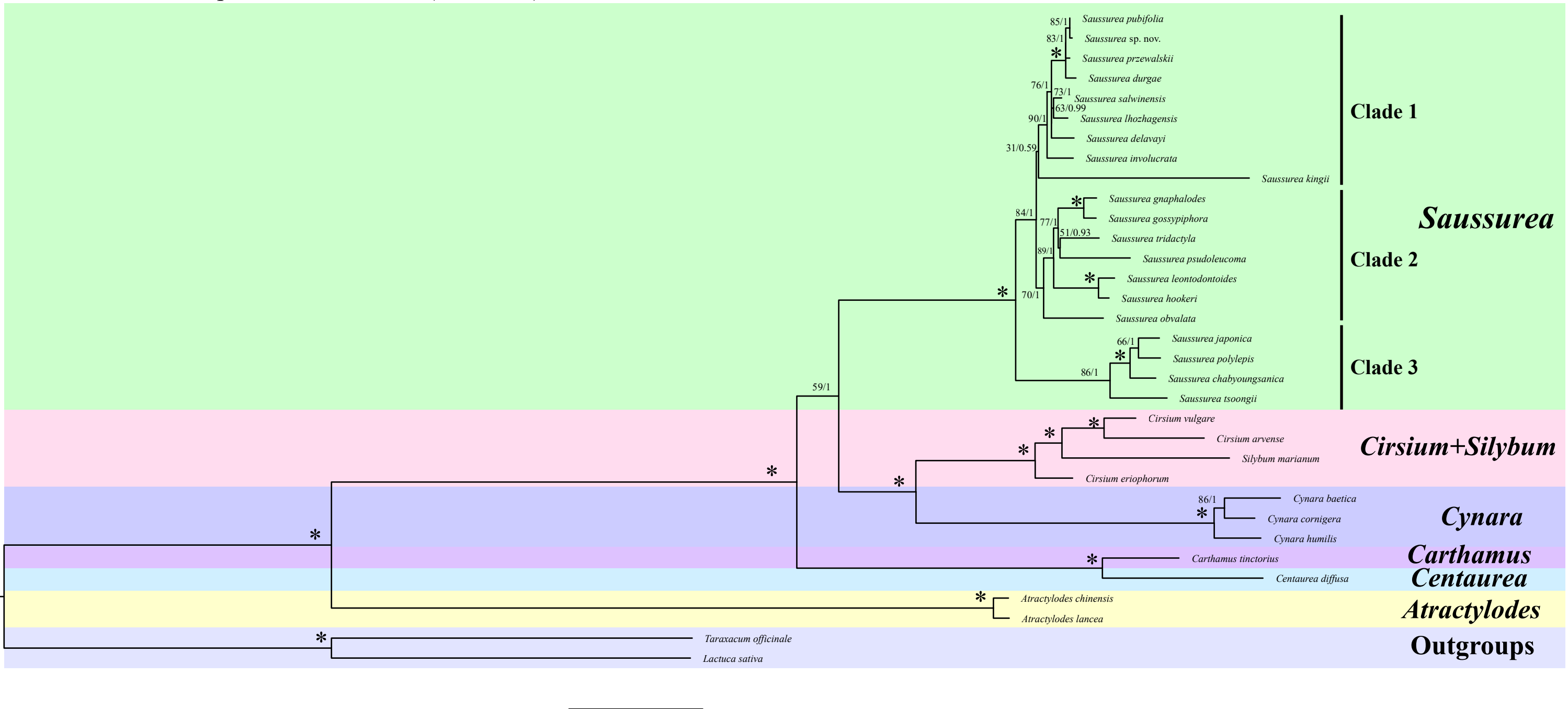

Supplement: Supplementary file 3 — Figure S2. Inferred molecular phylogeny from ML (maximum likelihood) and BI (Bayesian inference) analyses for the amino acid sequence of (79 CDS; data set 2). Maximum likelihood bootstrap values (BS) and posterior probabilities (PP) are shown at nodes. Branches with * have 100% bootstrap support and 1.0 posterior probability. (PDF 272 kb) [file 12870_2019_1896_MOESM3_ESM.pdf]

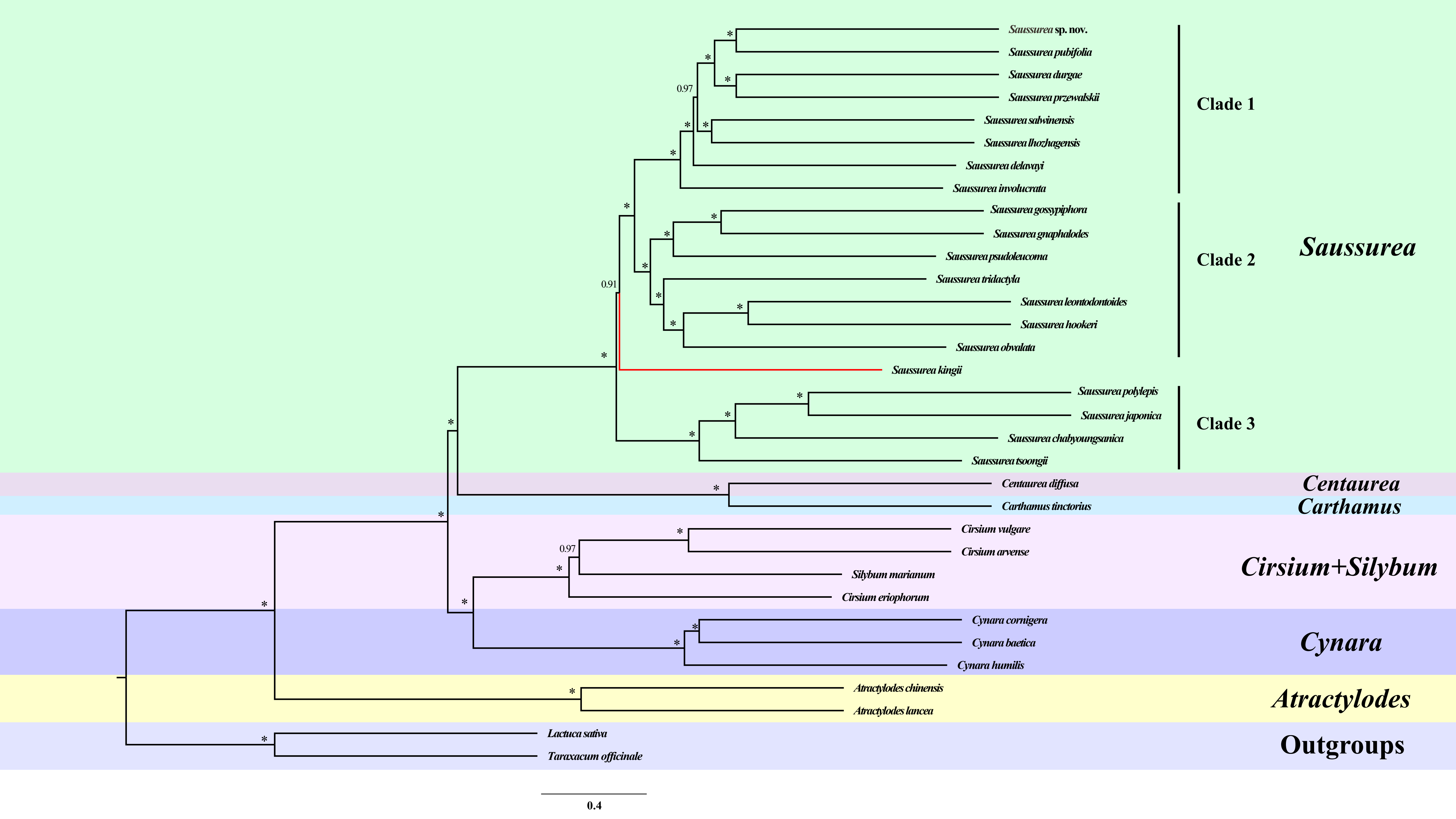

Supplement: Supplementary file 4 — Figure S3. Estimated species tree from 79 CDS alignment by coalescent-based approach. Local posterior probabilities are labeled at nodes. Branches with * have 1.0 posterior probability. The clade of S. kingii is colored in red. (PDF 548 kb) [file 12870_2019_1896_MOESM4_ESM.pdf]
